# Catalpol Ameliorates Podocyte Injury by Stabilizing Cytoskeleton and Enhancing Autophagy in Diabetic Nephropathy

**DOI:** 10.3389/fphar.2019.01477

**Published:** 2019-12-10

**Authors:** Yan Chen, Qingpu Liu, Zengfu Shan, Wangyang Mi, Yingying Zhao, Meng Li, Baiyan Wang, Xiaoke Zheng, Weisheng Feng

**Affiliations:** ^1^College of Pharmacy, Henan University of Chinese Medicine, Zhengzhou, China; ^2^College of Basic Medicine, Henan University of Chinese Medicine, Zhengzhou, China; ^3^Co-Construction Collaborative Innovation Center for Chinese Medicine and Respiratory Diseases by Henan & Education Ministry of P.R. China, Henan University of Chinese Medicine, Zhengzhou, China

**Keywords:** catalpol, diabetic nephropathy, podocyte injury, autophagy, cytoskeleton

## Abstract

Catalpol, an iridoid glycoside extracted from *Rehmannia glutinosa*, has been found to ameliorate diabetic nephropathy (DN), but the mechanism has not been clarified. Podocyte injury play a key role in the pathogenesis of DN. This study mainly investigated the protective effect and potential mechanism of catalpol on podocyte injury of DN *in vivo* and *in vitro*. The results indicated that the pathological features of DN in mice were markedly ameliorated after treatment with catalpol. Moreover, podocyte foot process effacement, and down-regulation of nephrin and synaptopodin expression in DN mice were also significantly improved after treatment with catalpol. *In vitro*, catalpol rescued disrupted cytoskeleton and increased migration ratio in podocytes induced by high glucose, the effect might be attributable to the inhibition of RhoA and Cdc42 activities but not Rac1. Furthermore, the impaired podocyte autophagy in DN mice was significantly enhanced after catalpol treatment. And catalpol also enhanced autophagy and lysosome biogenesis in cultured podocytes under high glucose condition. In addition, we found that catalpol could inhibit mTOR activity and promote TFEB nuclear translocation *in vivo* and *in vitro* experiments. Our study demonstrated that catalpol could ameliorate podocyte injury in DN, and the protective effect of catalpol might be attributed to the stabilization of podocyte cytoskeleton and the improvement of impaired podocyte autophagy.

## Introduction

Diabetic nephropathy (DN) is a common diabetic microvascular complication, which has been one of the leading causes of end-stage renal disease and increases the risks of cardiovascular disease events and death (Georgianos and Agarwal, 2017; Cho et al., 2018; Ravindran et al., 2019). Intensive glycemia control and treatments for hypertension are established therapies toward DN (Fineberg et al., 2013), despite proven effective therapies, they are not enough to arrest the development of DN to end-stage renal disease and there is, thus, the great need to develop additional therapies.

Albuminuria is the common clinically manifestation of DN that usually develops before the glomerular filtration rate (GFR) impaired and increases the risk of decline of GFR (Molitch et al., 2010), the prevention of albuminuria is therefore a recommendatory therapy in patients with DN (Looker et al., 2019). Podocyte play a key role in the pathogenesis of DN (Denhez et al., 2019). Multiple clinical studies have confirmed that podocyte foot process effacement is found in DN patients, and such podocyte injury is accompanied with abnormal glomerular filtration function (Shankland, 2006; George et al., 2012; Hara et al., 2019). As highly differentiated cells, podocytes line the urinary side of the glomerular basement membrane, which are essential for maintenance of the structure and function of the glomerular filtration barrier through their interdigitating foot processes (Nagata, 2016). This unique function of podocytes depends on their complex cytoskeleton structure, particularly the actin-rich foot processes. Dynamic regulation of the podocyte foot process cytoskeleton plays a critical role in maintaining sustained glomerular filter function, and podocyte injury is accompanied by the cytoskeleton rearrangement, which is closely associated with foot process effacement and subsequent proteinuria (Tryggvason et al., 2006; Wiggins, 2007; Jefferson et al., 2011; Toffoli et al., 2018). Given the key role of cytoskeleton dynamics, several previous studies have revealed that improving cytoskeleton dynamics would ameliorate DN (Kikuchi et al., 2007; Reiser and Sever, 2013; Lin and Susztak, 2016).

Autophagy is a highly conserved intracellular catabolic process for degradation of proteins and organelles *via* the lysosomal pathway (Mizushima and Komatsu, 2011; Mizushima et al., 2008). As terminally differentiated cells, podocytes exhibit a high level of basal autophagy, which play a pivotal role for maintaining podocyte's homeostasis (Hartleben et al., 2010; Yasuda-Yamahara et al., 2015; Jin et al., 2019). Previous studies have shown that podocyte autophagy insufficiency is found in diabetic patients accompanied by massive proteinuria, and impaired podocyte autophagy exacerbated proteinuria in DN, these studies indicate the importance of podocyte autophagy in the pathogenesis of DN (Kume and Koya, 2015; Lenoir et al., 2015; Tagawa et al., 2016). Recently, transcription factor EB (TFEB) was identified to regulated the transcription of various genes involved in autophagy and lysosomal biogenesis, and inhibition of the mammalian target of rapamycin (mTOR) has been shown to protect podocyte injury by promoting nuclear translocation of TFEB in animal models of DN (Settembre et al.,2011; Settembre et al., 2013).

**Figure 1:**
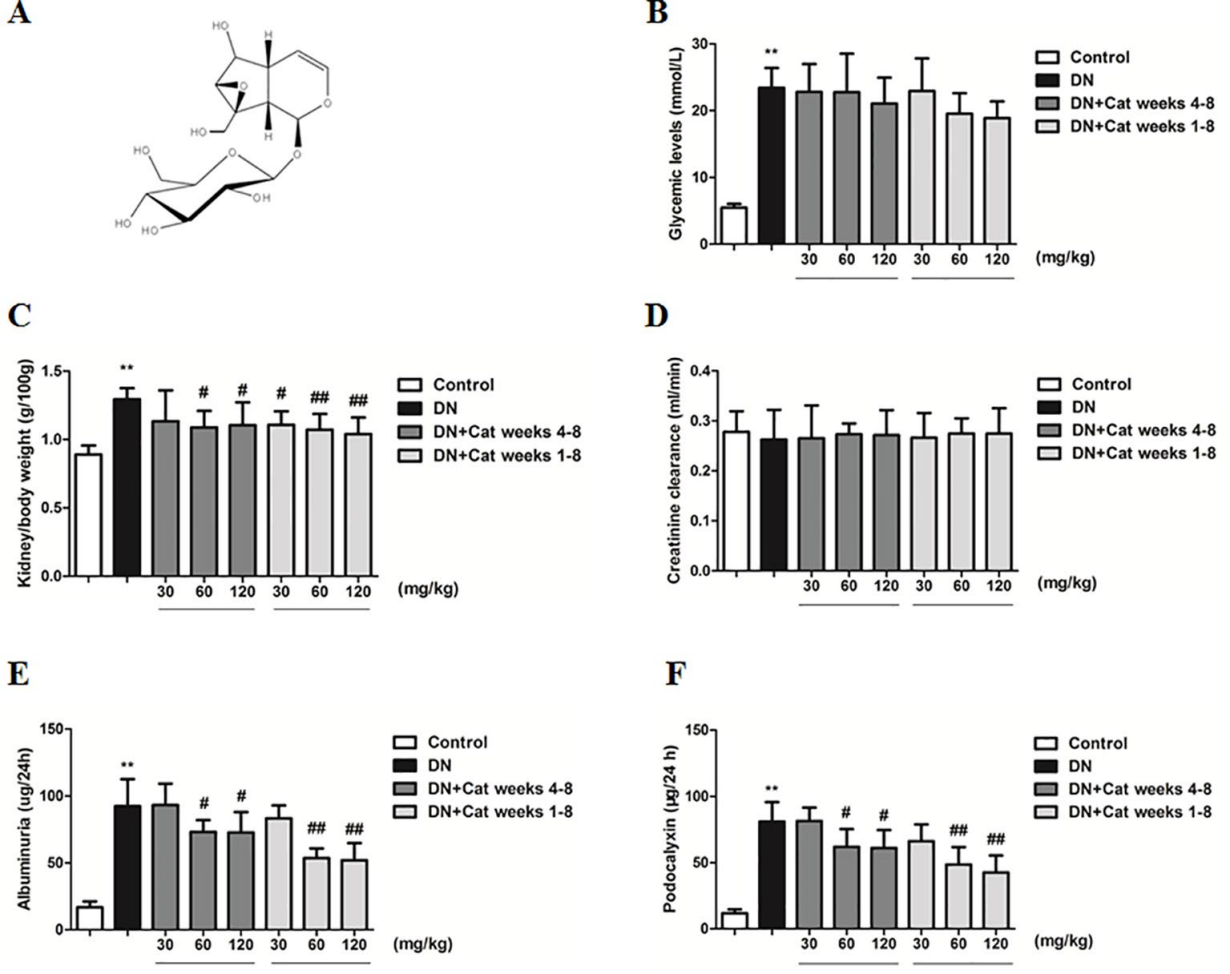
Effects of catalpol on physiological and renal functional parameters in diabetic nephropathy (DN) mice. **(A)** Chemical structure of catalpol. **(B)** The level of blood glucose in Control, DN, and DN plus catalpol-treated (30, 60, and 120 mg/kg, weeks 4–8 and weeks 1–8) mice (n = 8). **(C)** Kidney/body weight in Control, DN, and DN plus different doses of catalpol-treated mice (n = 8). **(D)** Creatinine clearance in Control, DN, and DN plus different doses of catalpol-treated mice (n = 8). **(E)** Urinary albumin excretion in Control, DN, and DN plus different doses of catalpol-treated mice (n = 8). (**F**) Urinary podocalyxin level in Control, DN, and DN plus different doses of catalpol-treated mice (n = 8). Data represent the mean values ± SD. **p < 0.01 vs Control, *^#^* *p* < 0.05, *^##^* *p* < 0.01 vs DN.

Catalpol (**Figure 1A**) is a natural iridoid glycoside compound derived from traditional Chinese medicinal herb *Rehmannia glutinosa*. It has been demonstrated that catalpol possesses a wide range of biological activities, including anti-diabetic, anti-inflammatory and neuroprotective effect (Chen et al., 2013; Liu et al., 2018; Yan et al., 2018). Additionally, previous study reported that catalpol could ameliorate renal function and proteinuria, which was closely associated with podocyte injury in DN (Dong and Chen, 2013). However, it is unclear whether catalpol exhibits a beneficial effect on podocyte injury in DN. In the present study, we investigated the effect and mechanism of catalpol on podocyte injury in DN, and found that catalpol could ameliorate podocyte injury through stabilizing cytoskeleton and enhancing autophagy in DN.

## Materials and Methods

### Materials

Catalpol (98%) was purchased from Nanjing Spring & Autumn Biological Engineering Co., Ltd. (Jiangsu, China). Primary antibodies directed against the following proteins were used: Anti-synaptopodin (sc-515842) was purchased from Santa Cruz Biotechnology (CA, United States). Anti-nephrin (ab216341), anti-RhoA (ab187027), anti-Cdc42 (ab187643), anti-Rac1 (ab180683), and anti-LC3B (ab48394) antibodies were purchased from Abcam (Cambridge, MA, USA). Anti-p62 (#23214), anti-p70s6k (#9202), anti-phospho-p70s6k (#9234), and anti-histone H3 (#4499) antibodies were purchased from Cell Signaling Technology (Danvers, MA, USA). Anti-TFEB (A303-673A) was purchased from Bethyl Laboratories, Inc (Montgomery, MA, USA). Anti-β-actin (AC026) was purchased from Abclonal (Boston, MA, USA).

### Animals Experimental Design

Male C57BL/6J mice (8 weeks) were obtained from the Beijing Vital River Laboratory Animal Technology Co., Ltd. (Beijing, China). Mice were housed in the central animal facility of the Henan University of Chinese Medicine and maintained on a normal diet under standard animal housing condition (temperature 25 ± 1°C and humidity 50% ± 10% with a 12-h dark/light cycle). After 7 days of acclimation, mice were intraperitoneal injection with streptozotocin in citrate buffer pH 4.5 at a dose 170 mg/kg of body weight to establish the diabetic model. Catalpol (Cat) (30, 60, 120 mg/kg) or vehicle was given by gavage once a day from weeks 4–8 or weeks 1–8 after streptozotocin administration (n = 8). Control non-diabetic mice were administered vehicle daily. All treatments continued for 8 weeks. At the end of experiment, the mice were anesthetized with 1.5% (w/v) pentobarbital sodium solution, then kidney tissues and blood samples were collected for further experiments. All animal experiments were approved by the Institutional Animal Care and Research Ethics Committee of Henan University of Chinese Medicine and confirmed to the guidelines of the National Institute of Health for the Care and Use of Laboratory Animals.

### Cell Culture

Conditionally immortalized mouse podocytes were provided by National Infrastructure of Cell Line Resource (Beijing, China) and described in detail previously (Mundel et al., 1997). Podocytes were cultured in RPMI 1640 medium (Life Technologies, Grand Island, NY) supplemented with 10% fetal bovine serum, 100 µg/ml streptomycin, and 100 U/ml penicillin. Recombinant mouse interferon-γ (50 U/ml, PeproTech, California, USA) was added to culture medium at 33°C in a humidified atmosphere of 5% CO_2_. To induce differentiation, podocytes were cultured in RPMI 1640 medium without IFN-γ at 37°C for 14 days. When podocytes were well-differentiated, they were incubated with normal glucose (NG) media (5.5 mmol/L glucose + 34.5 mmol/L mannitol) or high glucose (HG) media (40 mmol/L glucose) with or without catalpol (1, 5, 10 µmol/L) for 48 h and collected for the following assays.

### Physiological Parameters

Fasting blood glucose levels were measured by using a Glucometer (OMRON Corporation, Tokyo, Japanese). For urine collection, mice were held in a metabolic cage for 24 h. Levels of urinary albuminuria and podocalyxin were measured using ELISA kits (Elabscience, Wuhan, China). Creatinine was tested by a commercial assay kit (Jiancheng, Jiangsu, China).

### Histology and Immunohistochemistry

Kidney tissues from mice were fixed in 4% buffered paraformaldehyde for 2 days, embedded in paraffin, and processed for sectioning. Kidney sections were stained *via* periodic acid-Schiff staining (PAS), mesangial area was analyzed from digital pictures of 20–25 glomeruli per group using Image-Pro plus software. For immunohistochemistry, nephrin expression was detected on 3-µm paraffin-embedded kidney sections. After incubated in citrate antigen retrieval solution, sections were incubated with H_2_O_2_ to inhibited the endogenous peroxidase activity. After blocking with BSA, the sections were incubated with anti-nephrin antibody overnight at 4°C, then exposed to secondary antibody, horseradish peroxidase-labeled swine anti-rabbit IgG (Servicebio, Wuhan, China) for 1 h, specific labeling of nephrin was performed with the DAB substrate kit (Servicebio, Wuhan, China). Immunohistochemical quantification was expressed as the percentage of positive staining area using Image-Pro plus software.

### Transmission Electron Microscopy

Small fragments of renal cortex were fixed in glutaraldehyde and osmic acid, dehydrated with graded ethanol, and embedded in ethoxyline resin. Sections 70 to 80 nm thick were cut and viewed under transmission electron microscopy (TEM) (JEM-1400, JEOL, Japanese).

### Immunofluorescence

For immunofluorescence, fixed podocytes or paraffin-embedded kidney sections were labeled with primary antibody against synaptopodin or TFEB and then incubated with an FITC-conjugated anti-mouse antibody or AlexaFluor555-conjugated anti-rabbit antibody (Beyotime, Shanghai, China). F-actin was labeled with rhodamine phalloidin (Cytoskeleton Inc., Denver, USA). The slides were viewed using a confocal microscope (Olympus Corporation, Tokyo, Japan).

### Podocyte Migration Assays

After different treatments, cultured podocytes were scratched with a pipette. Images of the wounded area were taken on an inverted microscope at indicated time points and were analyzed using Image J (NIH, Bethesda, MD). The percentage of cell migration area was calculated as Area 0 h - Area 24 h/Area 0 h. RTCA xCELLigence system (Roche Applied Science) was used to real-time migration assay according to the manufacturer's instruction. Briefly, at the beginning, the lower chamber was filled with 10% fetal bovine serum and different treatments (NG, HG, or HG with different concentrations of catalpol), and 5 × 10^4^ cells were plated in the upper chamber with serum-free media and different treatments. The data were exported from RTCA software.

### Pull-Down Assay for Small GTPases Activities

RhoA, Cdc42, and Rac1 activities were determined by measuring Rhoketin RBD beads or PAK-PBD beads pulled down by GTP-RhoA and GTP-Cdc42/Rac1, respectively (Garcia-Mata et al., 2006). RhoA, Cdc42, and Rac1 were separated on 12% SDS-PAGE following the manufacturer's instructions (Cytoskeleton Inc., Denver, USA).

### Western Blot

The proteins from prepared renal cortex or cultured podocytes were quantified with BCA protein Assay kit (Solarbio Life Sciences, Beijing, China). Proteins were separated by 8%–15% SDS-PAGE and transferred onto polyvinylidene difluoride membrane. After blocking with 5% non-fat milk for 1 h, the membranes were incubated with primary antibodies against synaptopodin (1:500 dilution), RhoA (1:2000 dilution), Cdc42 (1:5000 dilution), Rac1 (1:1000 dilution), LC3B (1:1000 dilution), p62 (1:1000 dilution), p-p70s6k (1:1000 dilution), p70s6k (1:1000 dilution), TFEB (1:1000 dilution), histone H3 (1:1000 dilution), and β-actin (1:5000 dilution) overnight at 4°C. After being washed five times in TBST, the membranes were treated for 1 h with IRDyeIgG (1:5000 dilution) and washed five times in TBST again. Finally, the protein bands were visualized by Odyssey Infrared Imager (LI-COR Biosciences, USA) and quantified by using Image Studio software. The protein levels were normalized against the β-actin or histone H3.

### Transfection of Adenovirus

mRFP-GFP-LC3B adenovirus was used to detect the autophagy in cultured podocytes (Ginet et al., 2014). In brief, after incubating with mRFP-GFP-LC3B adenovirus for 6 h in RPMI 1640 medium, the medium was removed and replaced to NG medium (5.5 mmol/L glucose + 34.5 mmol/L mannitol) or HG medium (40 mmol/L glucose) with or without catalpol (1, 5, 10, µmol/L). Confocal images were acquired after 48 h treatment as described in podocyte culture using FV1200 biological confocal laser scanning microscope (Olympus Corporation, Japanese).

### Data Analysis

Statistical analyses were performed by using GraphPad Prism 5.0 software (GraphPad Software^®^, San Diego, CA, USA). The results were presented as mean ± SD. Statistical significance was assessed by one-way ANOVA followed by Dunnett's *post hoc* test. Differences with *p* < 0.05 was considered as significant.

## Results

### Effects of Catalpol on Physiological and Renal Functional Parameters in DN Mice

As shown in **Figure 1B**, the level of blood glucose was significantly higher in DN compared to control mice, treatment with catalpol did not reverse the change. The kidney/body weight ratio in DN mice was significantly increased compared to that in control mice, treatment with catalpol at doses of 60 and 120 mg/kg (weeks 4–8) or at doses of 30, 60, and 120 mg/kg (weeks 1–8) significantly decreased the kidney/body weight ratio (**Figure 1C**). There was only a non-significant decline in creatinine clearance in DN mice compared to control mice (**Figure 1D**). At the end of the treatment period, urinary albumin excretion was significantly increased in DN mice compared to control mice, treatment with catalpol at doses of 60 and 120 mg/kg (weeks 4–8 and weeks 1–8) significantly reduced urinary albumin excretion (**Figure 1E**). In accordance with the results on albuminuria, DN mice exhibited a significant increase in the level of podocalyxin in urine compared to control mice, treatment with catalpol at doses of 60 and 120 mg/kg (weeks 4–8 and weeks 1–8) attenuated the increase in the level of podocalyxin in urine (**Figure 1F**).

### Effect of Catalpol on Renal Histological Changes in DN Mice

**Figure 2:**
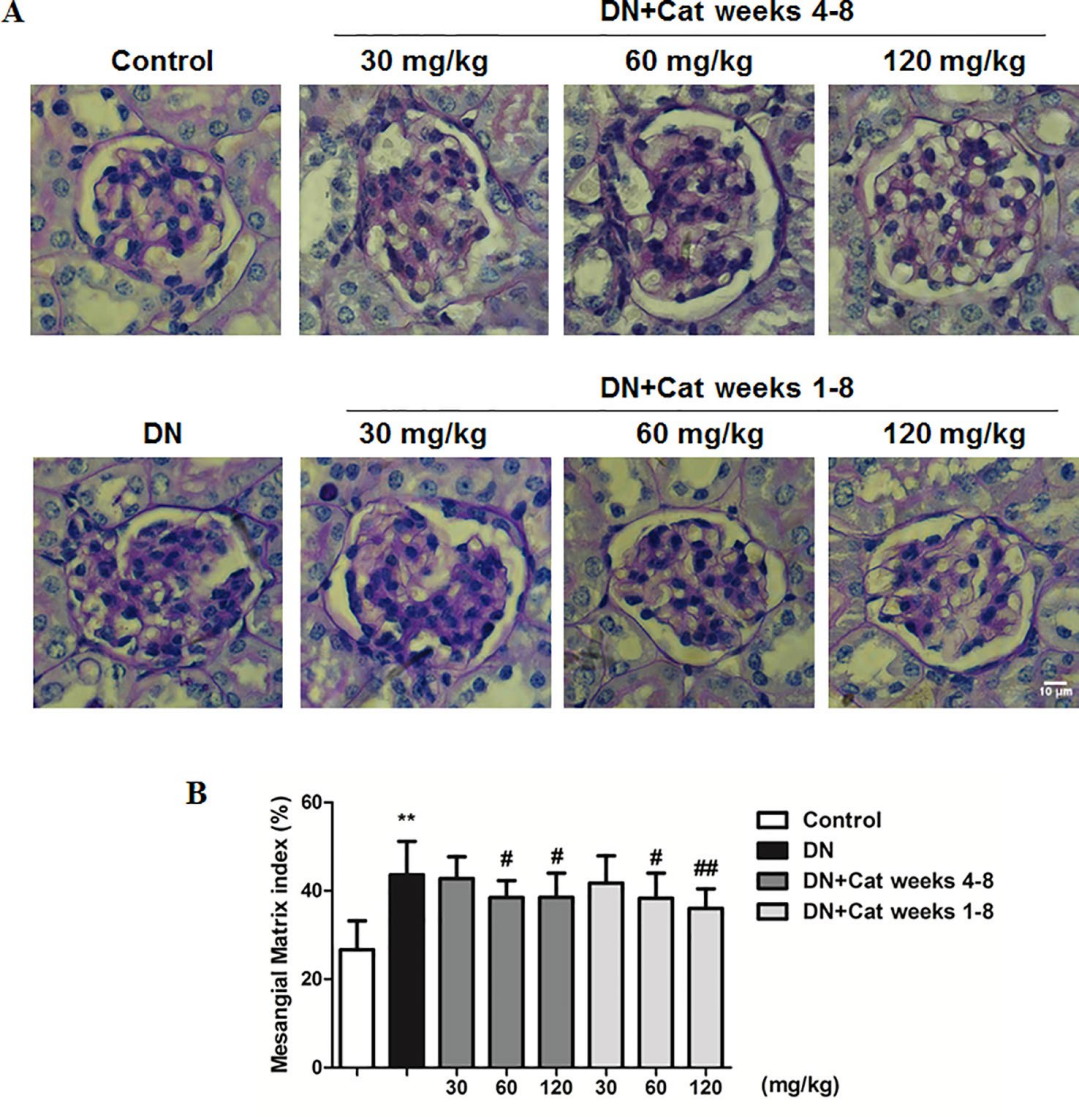
Effect of catalpol on renal histological changes in DN mice. **(A)** Representative images of periodic acid-Schiff (PAS) staining in glomeruli from Control, DN, and DN plus catalpol-treated (30, 60, and 120 mg/kg, weeks 4–8 and weeks 1–8) mice (n = 8). Scale bar: 10 µm. **(B)** Percentage of PAS glomerular-positive areas from Control, DN, and DN plus different doses of catalpol (n = 8, 20–25 images from each group). Data represent the mean values ± SD. **p < 0.01 vs Control, *^#^* *p* < 0.05, *^##^* *p* < 0.01 vs DN.

Histological changes in DN patients were characterized by glomerular hypertrophy and mesangial expansion (Li et al., 2007). Kidney PAS staining showed that DN mice revealed remarkable mesangial expansion, treatment with catalpol at doses of 60 and 120 mg/kg (weeks 4–8 and weeks 1–8) had a significant decrease in PAS–positive matrix (**Figure 2**).

### Effect of Catalpol on Podocyte Injury in DN Mice

**Figure 3:**
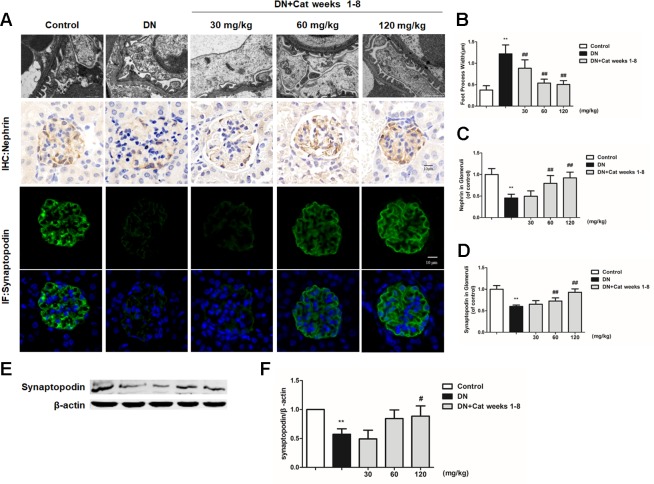
Effect of catalpol on podocyte injury in DN mice. **(A)** Representative transmission electron microscopy (TEM) images of podocyte foot processes in Control, DN, and DN plus catalpol-treated (30, 60, and 120 mg/kg, weeks 1–8) mice (n = 3). Scale bar: 1 µm. Representative images of glomeruli showing nephrin-positive and synaptopodin-positive expression in glomeruli of Control, DN, and DN plus different doses of catalpol-treated mice (n = 8). Scale bar: 10 µm. **(B)** Quantification of podocyte foot process width by electron micrographs (n = 3, 10 images from each group). **(C)** Quantification of results for immunohistochemical staining of nephrin expressed as the percentage of stained area in glomeruli (n = 8, 20–25 images from each group). **(D)** Quantification of results in synaptopodin in glomeruli by the ratio of integrated optical density (IOD) to area (n = 8, 21–25 images from each group). **(E)** Western blotting for expression of synaptopodin in Control, DN, and DN plus different doses of catalpol-treated mice (n = 3). **(F)** Quantification of results in panel E. Data represent the mean values ± SD. **p < 0.01 vs Control, ^#^ *p* < 0.05, *^##^* *p* < 0.01 vs DN.

Podocyte foot process effacement is considered as one of the early clinical manifestations, which strongly correlated with albuminuria and decreased GFR in DN patients (Lin and Susztak, 2016). As shown in **Figures 3A, B**, abnormal morphology and segmental effacement of podocyte foot processes were observed in DN mice by TEM, while treatment with catalpol at doses of 30, 60 and 120 mg/kg (weeks 1–8) could ameliorate foot processes effacement. Next, we measured podocyte injury by immunohistochemistry and immunofluorescence with nephrin and synaptopodin, two podocyte-specific markers. As shown in **Figures 3A, C, D**, there was a significant reduction in both nephrin-positive and synaptopodin-positive area in glomeruli of DN mice relative to control mice, the reduction was significantly ameliorated in catalpol-treated (60, 120 mg/kg) DN mice. In keeping with the results on immunofluorescence, the data were confirmed by measuring synaptopodin expression in renal cortex by western blotting (**Figures 3E, F**).

### Effects of Catalpol on Cytoskeleton and Migration in Podocytes Exposed to HG

**Figure 4:**
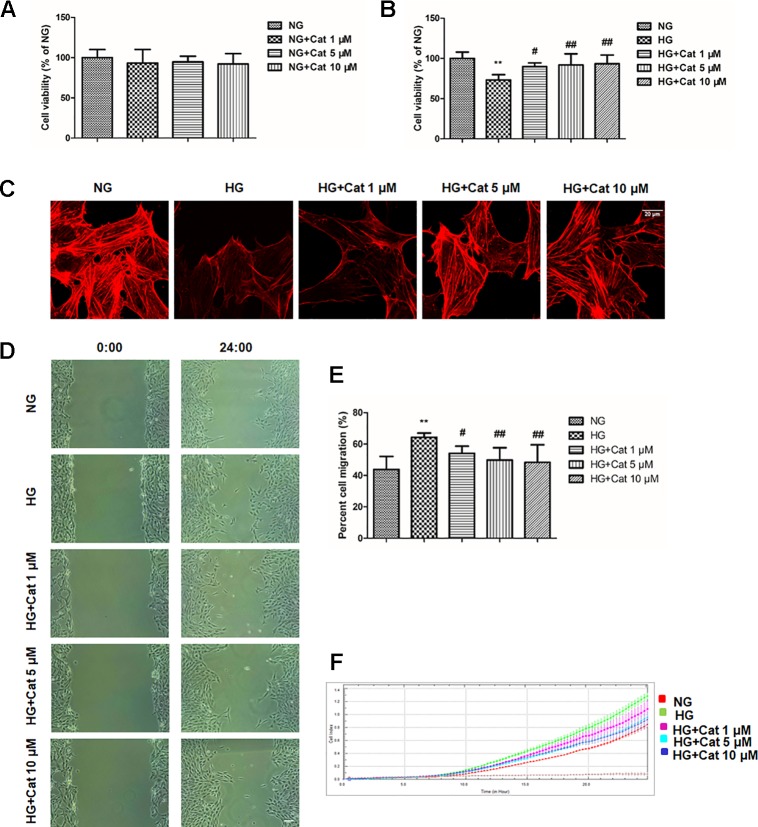
Effects of catalpol on cytoskeleton and migration in podocytes exposed to high glucose. **(A)** Cell viability was determined by MTT. Podocytes were cultured with normal glucose (NG) (5.5 mmol/L) and NG plus different concentrations of catalpol (1, 5, 10 μM) for 48 h. **(B)** Cell viability was determined by MTT. Podocytes were cultured with NG (5.5 mmol/L), high glucose (HG) (40 mmol/L), and HG plus different concentrations of catalpol (1, 5, 10 μM) for 48 h. **(C)** Rhodamine phalloidin staining of podocytes. Scale bar: 20 µm. **(D)** The images were recorded immediately (0 h) and at 24 h after scratch (n = 3). Scale bar: 200 µm. **(E)** Quantification of the cell migration area. (n = 3, 10 areas from each group). **(F)** Migration assay using the RTCA xCELLigence system (detailed in *Materials and Methods*). Data represent the mean values ± SD, **p < 0.01 vs NG, *^#^* *p* < 0.05, *^##^* *p* < 0.01 vs HG.

Podocytes were treated with different concentrations (1, 5, 10 µM) of catalpol in NG or HG medium in the MTT test. As shown in **Figure 4A**, treatment with catalpol did not alter cell viability compared to that of podocytes treated with NG. HG treatment significantly decreased cell viability in podocytes compared to that in cells treated with NG, however, treatment with catalpol significantly increased cell viability in podocytes incubated with HG (**Figure 4B**).

Cytoskeletal structure is critical to podocyte foot processes and is essential for maintaining the integrity of the glomerular filtration barrier (Lal and Patrakka, 2018), we next observed cytoskeletons using rhodamine phalloidin staining by confocal microscope. As expected, exposure of cultured podocytes to HG significantly disrupted podocyte F-actin stress fiber cytoskeletal structure, treatment with catalpol at concentrations of 5 and 10 µM could rescue the disruption of F-actin stress fiber in podocytes exposed to HG (**Figure 4C**).

Then, we detected the effect of catalpol on podocyte migration using wound healing assay and RTCA. As shown in **Figures 4D, E**, wound healing assay showed that podocytes with high concentration of glucose showed a significantly accelerated wound healing kinetics compared to NG group, the migration rates of podocytes were remarkably decreased by treatment with catalpol at concentrations of 1, 5 and 10 µM. The RTCA experiment showed that catalpol treatment reversed the increased migration ratio induced by HG, which was in accordance with the wound healing assay (**Figure 4F**).

### Effect of Catalpol on the Activities of RhoA, Cdc42, and Rac1 in Podocytes Exposed to HG

**Figure 5:**
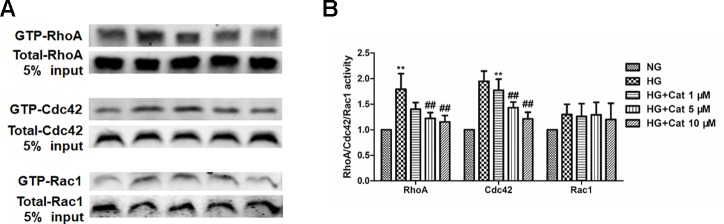
Effects of catalpol on the activities of RhoA, Cdc42, and Rac1 in podocytes exposed to high glucose. **(A)** Active GTP-bound forms of RhoA, Cdc42, and Rac1 purified from podocytes cultured with NG (5.5 mmol/L), HG (40 mmol/L), and HG plus different concentrations of catalpol (1, 5, 10 μM) by GST pull-down assays and subjected to western blotting (n = 3). **(B)** Quantification of results in panel A. Data represent the mean values ± SD from three independent experiments, **p < 0.01 vs NG, *^##^* *p* < 0.01 vs HG.

Given that the pivotal role of Rho family of GTPases in cytoskeletal dynamics, we further detected the activities of RhoA, Cdc42, and Rac1 in culture podocytes by GST pull-down assay (Huang et al., 2018; Pan et al., 2018). Our results revealed no significant difference in the Rac1 activation levels between NG and HG group but a notable augmentation of the RhoA-GTP level and Cdc42-GTP level in the HG group compared with those in the NG group, treatment with catalpol at doses of 5 and 10 µM inhibited the RhoA and Cdc42 activities, suggesting that catalpol maintained stable podocyte cytoskeleton through controlling RhoA and Cdc42 activities (**Figure 5**).

### Effect of Catalpol on Podocyte Autophagy in DN Mice

**Figure 6:**
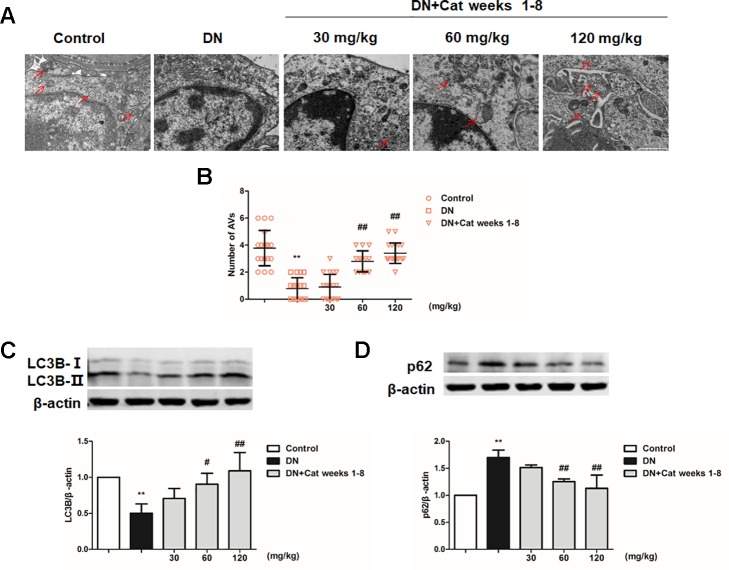
Effect of catalpol on podocyte autophagy in DN mice. (A) Representative TEM images of autophagic vacuoles (red arrow) in Control, DN, and DN plus catalpol-treated (30, 60, and 120 mg/kg, weeks 1–8) mice (n = 3). Scale bar: 1 µm. **(B)** The numbers of autophagic vacuoles by electron micrographs (n = 3, 15–20 electron micrographs were selected from each group). **(C**–**D)** Western blotting for expression of LC3B **(C)** and p62 **(D)** from the renal cortex in Control, DN, and DN plus different doses of catalpol-treated mice (n = 3). Data represent the mean values ± SD from three independent experiments, **p < 0.01 vs Control, *^#^* *p* < 0.05, *^##^* *p* < 0.01 vs DN.

To explore podocyte autophagy in DN mice, we examined the number of autophagic vacuoles in podocytes by TEM. We observed that the number of autophagic vacuoles was significantly decrease in DN mice compared to control mice, treatment with catalpol at doses of 60 and 120 mg/kg (weeks 1–8) significantly increased the number of autophagic vacuoles (**Figures 6A, B**). p62, a selective substrate of autophagy, which was found abnormal accumulation in the glomeruli of DN patients (Tagawa et al., 2016; Lin et al., 2019). There was a significant decrease in the autophagy marker LC3B expression and increase in p62 expression of DN mice which was ameliorated by catalpol treatment at doses of 60 and 120 mg/kg (**Figures 6C, D**).

### Effect of Catalpol on Autophagy in Podocytes Exposed to HG

**Figure 7:**
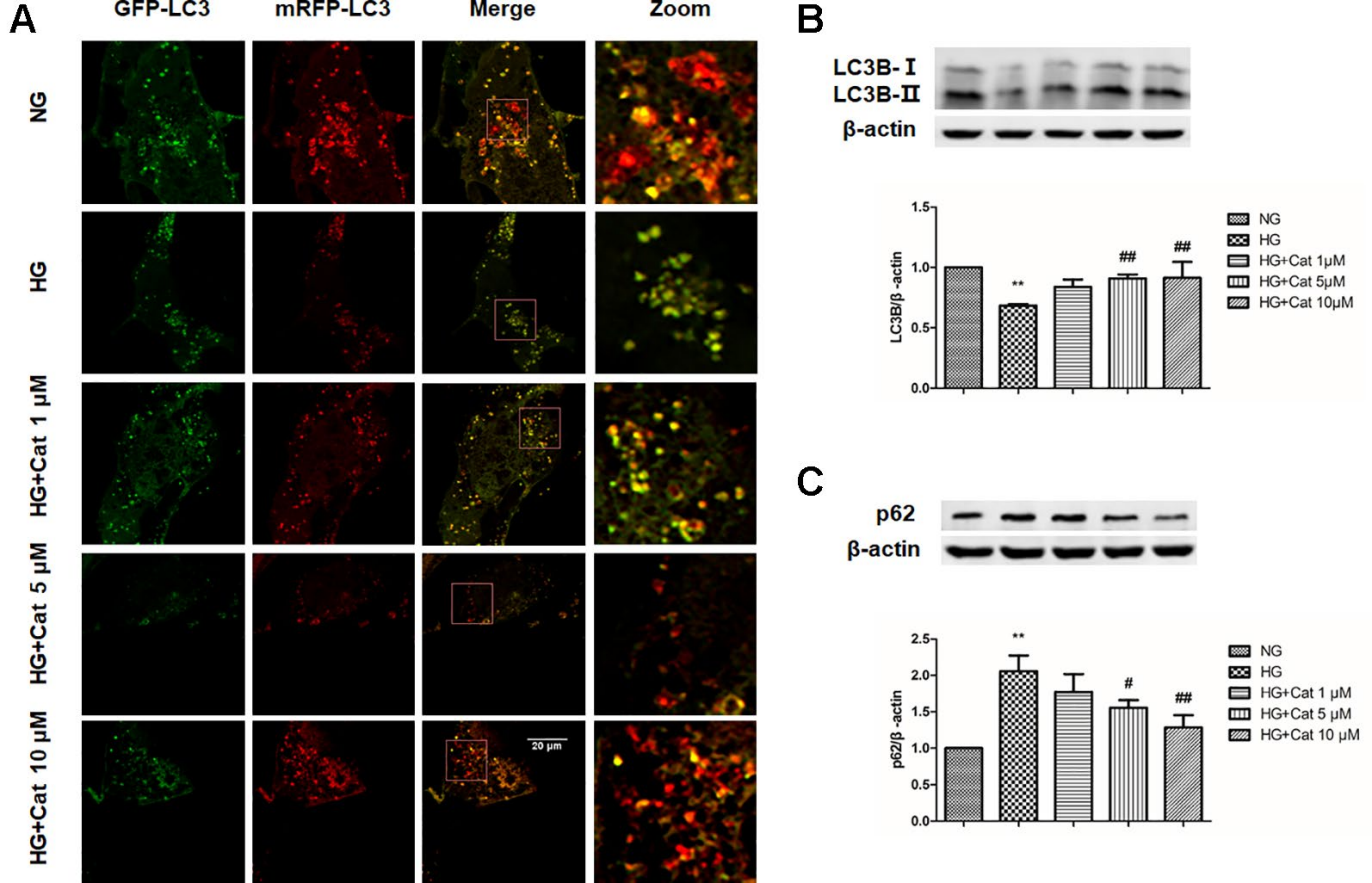
Effect of catalpol on autophagy in podocytes exposed to high glucose. **(A)** Confocal laser scanning microscopy images of podocytes cultured with NG (5.5 mmol/L), HG (40 mmol/L), and HG plus different concentrations of catalpol (1, 5, 10 μM). Scale bar: 20 µm. **(B**–**C)** Western blotting for expression of LC3B **(B)** and p62 **(C)** from cultured podocytes in NG (5.5 mmol/L), HG (40 mmol/L), and HG plus different concentrations of catalpol (1, 5, 10 μM) (n = 3). Data represent the mean values ± SD, **p < 0.01 vs NG, *^#^* *p* < 0.05, *^##^* *p* < 0.01 vs HG.

In Ad-mRFP-GFP-LC3B-transfected podocytes, high concentration of glucose induced the decrease of mRFP fluorescence, indicating autophagy insufficiency, in contrast, treatment with catalpol (10 µM) increased the number of mRFP fluorescence puncta, indicating the enhancing autophagy (**Figure 7A**). Furthermore, the decreased LC3B expression and increased p62 expression induced by HG were remarkably reversed by treatment with catalpol at concentrations of 5 and 10 µM (**Figures 7B, C**).

### Effects of Catalpol on mTOR/TFEB Pathway in DN Mice and Podocytes Exposed to HG

**Figure 8:**
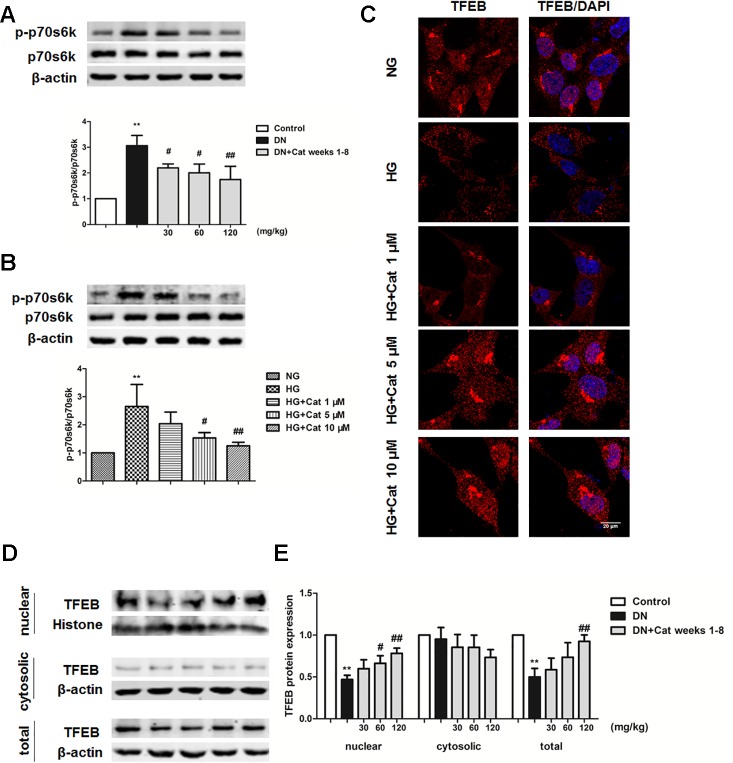
Effects of catalpol on mTOR/TFEB pathway in DN mice and podocytes exposed to high glucose. **(A)** Representative blots show the expression of phosphorylated and total p70S6K in Control, DN, and DN plus catalpol-treated (30, 60, and 120 mg/kg, weeks 1–8) mice (n = 3). **(B)** Representative blots show the expression of phosphorylated and total p70s6k in podocytes cultured with NG (5.5 mmol/L), HG (40 mmol/L), and HG plus different concentrations of catalpol (1, 5, 10 μM) (n = 3). **(C)** Representative images of podocytes stained with TFEB antibody (red) and DAPI (blue). Scale bar: 20 µm. **(D)** Blots of the nuclear, cytosolic, and total TFEB expression in Control, DN, and DN plus different doses of catalpol-treated mice (n = 3). **(E)** Quantification of results in panel D. Data represent the mean values ± SD from three independent experiments, **p < 0.01 vs Control, *^#^* *p* < 0.05, *^##^* *p* < 0.01 vs DN.

To further explore the possible mechanism underlying the enhanced effect of catalpol on impaired podocyte autophagy, we determined mTOR activity and TFEB nuclear translocation in DN mice and cultured podocytes. As shown in **Figure 8A**, western blotting revealed that phosphorylated p70s6k (p-p70s6k) were significantly increased in DN mice compared with that in control mice, however, treatment with catalpol at doses of 30, 60, and 120 mg/kg (weeks 1–8) significantly decreased p-p70s6k expression. *In vitro*, exposure of cultured podocytes to HG induced a significant increase in p-p70s6k expression, treatment with catalpol at concentrations of 5 and 10 µM decreased the expression of p-p70s6k (**Figure 8B**). Since inhibition of mTOR activity could promote TFEB nuclear translocation (Settembre et al., 2012; Zhao et al., 2018; Ye et al., 2019), we next determined whether catalpol could activate TFEB and promote its nuclear translocation. As shown in **Figure 8C**, catalpol treatment at concentrations of 5 and 10 µM showed an obviously effect on TFEB nuclear translocation in cultured podocytes incubated with HG. *In vivo*, western blotting showed that treatment with catalpol (60, 120 mg/kg) significantly rescued the down-regulation of nucleus TFEB expression in DN mice, suggesting that catalpol promoted TFEB nuclear translocation in DN mice (**Figures 8D, E**).

## Discussion

Podocyte injury play a key role in the progression of DN (dai et al., 2017; Kim et al., 2018). In the present study, we provided evidence that in an animal model of DN, treatment with catalpol ameliorated podocyte injury by stabilizing podocyte cytoskeleton structure and rescuing podocyte autophagy in DN.

Albuminuria, an earliest marker of DN, which is related to podocyte injury in the progression of DN (Dinneen and Gerstein, 1997). Kidney hypertrophy, a common manifestation of DN patients, which is considered as an elevated kidney/body weight ratio (Bakris, 2008). In addition, glomerular mesangial expansion is another common pathological manifestation of DN. In this study, we demonstrated that treatment with catalpol significant decreased albuminuria levels and improved kidney hypertrophy and mesangial expansion, providing evidence of catalpol efficacy, and these results were consistent with previous study (Dong and Chen, 2013). Although it has been reported that catalpol lowered blood glucose in diabetes mice (Li et al., 2014), in our study, a modest and non-significant decrease in blood glucose was observed in catalpol-treated mice, and thus, the renoprotection of catalpol was independent of blood glucose. In addition, there was a modest decline in creatinine clearance in DN mice, precluding evaluation of treatment effect on this factor.

Several studies indicated that drugs have protective effects on podocyte injury could improve the ability to ameliorate DN (Zhang et al., 2013; Kim et al., 2018). In this study, treatment with catalpol lowered the excretion of urinary podocyte marker podocalyxin, suggesting the protective effect of catalpol on podocyte injury in DN. Clearly, we observed a significant improvement in podocyte foot process effacement in catalpol-treated DN mice by using TEM. Nephrin, which is identified at the slit diaphragm of podocyte and is critical to maintain the integrity of filtration barrier (Trohatou et al., 2017). Synaptopodin, a actin-binding protein, which is located at podocyte cytoskeleton (Pan et al., 2018). When podocytes undergo injury in DN, the expression of nephrin and synaptopodin were significantly decreased, our results showed that treatment with catalpol significantly increased the down-regulation of nephrin and synaptopodin in DN mice. These results suggested that the renoprotection of catalpol was likely to be attributed to the improvement of podocyte injury in DN.

Podocyte injury accompanied by cytoskeleton rearrangement is observed in DN. Cytoskeleton rearrangement was closely associated with foot process effacement and albuminuria in several kidney diseases such as DN (Giganti and Friederich, 2003; Faul et al., 2008). Our results showed that catalpol could improve cytoskeleton disruption induced by HG, suggesting that catalpol improved podocyte injury by regulating the dynamics of the cytoskeleton. Recently study showed that, when podocytes undergo foot process effacement, podocytes shifted to a dynamic state from non-motile state *in vivo* (Brähler et al., 2016). In our study, the results strongly argue that catalpol suppressed podocyte migration induced by HG. These data demonstrated that catalpol protect podocyte injury through regulating the dynamics of the cytoskeleton and decreasing the motility of podocyte. Rho family of GTPases, particularly RhoA, Cdc42, and Rac1 are important molecular switches, which are closely related to dynamics of podocyte cytoskeleton (Wang et al., 2012). It has been shown that Podocyte-specific Rac1 deficiency promoted foot process effacement and proteinuria by disrupting cytoskeleton in DN mice (Scott et al., 2012; Blattner et al., 2013; Lv et al., 2018). In addition, the excessive activation of RhoA in podocytes resulted in foot process effacement and heavy proteinuria, accompanied by podocyte cytoskeleton disruption (Zhu et al., 2011; Wang et al., 2012). Therefore, controlled RhoA, Cdc42, and Rac1 activities are vital to stable podocyte cytoskeletons. In this study, our data revealed that catalpol could inhibit the excessive activation of RhoA and Cdc42 in podocytes induced by HG, suggesting that catalpol stabilized podocyte cytoskeleton through modulating the active forms of RhoA and Cdc42 but not Rac1.

Moreover, insufficient autophagy was observed specifically in podocytes of DN patients, which aggravated podocyte injury and proteinuria in DN. Thus, enhancing autophagy in podocytes is a potential treatment strategy for DN (Lenoir et al., 2015; Tagawa et al., 2016). It has been reported that catalpol promoted autophagy in liver fibrosis rats (Liu et al., 2018). In the present study, autophagosome numbers and LC3B expression were decreased, and p62 expression was increased in DN mice when compared with control mice, suggesting podocyte autophagy insufficiency in DN mice. In HG cultured podocytes, the number of autolysosome and the autophagy marker LC3B expression were decreased, and the p62 expression was increased, indicating that the autophagy flux was blocked in podocytes incubated with HG, while treatment with catalpol could improve podocyte autophagy *in vivo* and *in vitro*. These results demonstrated that catalpol improved podocyte autophagy insufficiency in DN. Furthermore, the exceeding activation of mTOR has been reported in podocytes in DN patients, which play a key role in the progression of DN (Gödel et al., 2011; Inoki et al., 2011). Moreover, TFEB was considered as the master regulator of autophagy (Settembre et al., 2011). It has been reported that the nuclear translocation of TFEB is inhibited by mTOR activation, and inhibition of mTOR activity could promote TFEB nuclear translocation to protect podocyte injury in DN (Martina et al., 2012; Zhao et al., 2018). Our results suggested that mTOR was hyperactive, and accompanied with the decreased nuclear translocation of TFEB *in vivo* and *in vitro*, treatment with catalpol inhibited mTOR activity and promoted TFEB nuclear translocation in DN. These results suggested that catalpol enhanced podocyte autophagy through inhibiting mTOR activity to promote TFEB nuclear translocation, and subsequently enhanced autophagy.

## Conclusion

In conclusion, our study demonstrated that catalpol could improve podocyte injury in DN, and the protective effect of catalpol might be attributed to the stabilization of podocyte cytoskeleton structure and the improvement of impaired podocyte autophagy.

## Data Availability Statement

All datasets generated for this study are included in the article/supplementary material.

## Ethics Statement

All animal experiments were approved by the Institutional Animal Care and Research Ethics Committee of Henan University of Chinese Medicine and conformed to the guidelines of the National Institute of Health for the Care and Use of Laboratory Animals.

## Author Contributions

YC, QL, and XZ contributed to the experiment design and manuscript writing. YC, QL, ZS, WM, YZ, ML, and BW performed the experiments. YC and QL analyzed the data. XZ and WF supervised the project. All authors read and approved the final manuscript.

## Funding

This study was supported by The National Key Research and Development Project (The Major Project for Research of the Modernization of TCM): Key Technology Research for the Characteristic Chinese Medicine Industry Chain of *Rehmannia glutinosa* (2017YFC1702800) and The Major Science and Technology Projects in Henan Province: Study on the key technology for quality control and the key characteristics of *R. glutinosa*, *Dioscorea opposita* Thunb, and *Achyranthes bidentata* Blume from Henan Province (171100310500).

## Conflict of Interest

The authors declare that the research was conducted in the absence of any commercial or financial relationships that could be construed as a potential conflict of interest.
